# Left ventricular myocardial work in pregnant women with autoimmune diseases

**DOI:** 10.3389/fcvm.2026.1691607

**Published:** 2026-03-06

**Authors:** Lu Zhang, Yilu Shi, Yaxi Wang, Xiaoshan Zhang, Shasha Duan

**Affiliations:** Department of Ultrasound Diagnosis, Affiliated Hospital of Inner Mongolia Medical University, Hohhot, Inner Mongolia, China

**Keywords:** autoimmune diseases, echocardiography, left ventricular function, myocardial work, pregnancy

## Abstract

**Purpose:**

To quantitatively assess left ventricular (LV) myocardial work (MW) in pregnant women with autoimmune diseases (AD) using left ventricular pressure-strain loop (PSL) and explore its clinical implications.

**Methods:**

Ninety-six participants were enrolled between September 2020 and September 2022 at the Affiliated Hospital of Inner Mongolia Medical University, including 33 pregnant women with AD (AD-P group), 26 non-pregnant AD patients (AD group), and 37 healthy pregnant women (H-P group). Clinical data, conventional echocardiography, two-dimensional speckle-tracking, and LV-MW analyses were obtained. Group comparisons and correlations between baseline characteristics and MW parameters were analyzed. Analysis of covariance (ANCOVA) and partial correlation were used for adjusted comparisons and associations.

**Results:**

Following adjustment, the AD-P group demonstrated increased LV volume and lower apical constructive work (CW) compared to the AD group, while global MW indices were similar. Compared with H-P group, AD-P patients had lower E/A, increased LV volumes, E/e’, and peak strain dispersion (PSD). After adjustment, AD-P had reduced global work index (GWI), global constructive work (GCW), global work efficiency (GWE), and apical-CW, while PSD remained higher.

**Conclusion:**

LV myocardial work detected subclinical LV dysfunction in pregnant women with autoimmune disease. Apical-CW showed a consistent reduction in AD-P. These findings suggest that myocardial work, especially apical CW, provides incremental value over global longitudinal strain (GLS) in autoimmune pregnancies.

## 1 Introduction

Autoimmune diseases (AD) are characterized by systemic inflammation that leads to multi-organ injury and dysfunction. Cardiac involvement occurs in up to 50% of patients, and because early myocardial impairment is often lacks clinical specificity, cardiovascular disease has become a leading causes of late mortality in this population (1, 2). AD predominantly affect women of reproductive age, and pregnancy-related factors such as hormonal fluctuations and fetomaternal microchimerism may further modulate disease activity (3). Previous studies have shown that pregnant women with AD have a substantially higher prevalence of cardiovascular complications than healthy pregnant women, and these events tend to occur at a younger age (4). Whether the maternal cardiovascular system can adequately adapt to the hemodynamic demands of pregnancy is therefore critical for both maternal and fetal outcomes. Early and accurate assessment of cardiac function in pregnant women with AD is thus of considerable clinical importance, yet data remain limited.

Echocardiography is the preferred modality for cardiac assessment during pregnancy. In recent years, quantitative techniques for myocardial function have attracted increasing attention. Two-dimensional speckle-tracking echocardiography (2D-STE) allows quantitative evaluation of myocardial mechanics. Global longitudinal strain (GLS) derived from 2D-STE provides a more sensitive and comprehensive measure of global and regional left ventricular (LV) function than left ventricular ejection fraction (LVEF) and is useful for detecting subclinical myocardial dysfunction. However, its marked load dependence may compromise the accuracy of functional assessment in conditions with altered preload and afterload (5). Non-invasive left ventricular pressure-strain loop (PSL) analysis allows quantification of myocardial work (MW) to assess LV systolic performance (6). This method partially overcomes the load dependence of strain while maintaining sensitivity to myocardial injury and has been increasingly applied in a variety of cardiac diseases (7, 8).

Based on these considerations, the present study applied non-invasive LV-PSL to evaluate myocardial function in pregnant women with AD and to explore its clinical implications. We hypothesized that pregnancy-related volume load superimposed on chronic inflammation would be associated with impaired MW parameters despite preserved conventional parameters.

## 2 Materials and methods

### 2.1 Study design and population

This single-center, cross-sectional, and case-control study was conducted at the Affiliated Hospital of Inner Mongolia Medical University. Echocardiographic image acquisition and offline analyses were performed by readers blinded to clinical group assignment. All procedures complied with the Declaration of Helsinki, and written informed consent was obtained from all participants. The study protocol was approved by the institutional ethics committee [Approval No. WZ (2022052)].

A total of 111 participants were randomly enrolled, including mid-pregnancy women with autoimmune diseases (AD-P group) and non-pregnant patients with autoimmune diseases (AD group) from the Department of Rheumatology and Immunology, and healthy mid-pregnancy controls (H-P group) from the Department of Obstetrics and Gynecology.

### 2.2 Sample size calculation

The sample size was determined using data from our preliminary study, in which the global work efficiency (GWE) was our primary outcome. he expected group means were 96.6% in the AD-P group, 96.5% in the H-P group, and 95.0% in the AD group, with a common standard deviation of 1.66. Assuming a two-sided test with an alpha level of 0.05, a power of 85%, and equal allocation across the three groups, PASS 2021 software (procedure: Means→One-Way ANOVA, *F*-tests) indicated that 18 participants per group were required. Allowing for an anticipated 20% attrition rate, the target sample size was set at ≥22 participants per group.

### 2.3 Inclusion and exclusion criteria

In the AD-P group, forty pregnant women with AD in the second trimester were initially screened between September 2020 and September 2022. Seven were excluded (one twin pregnancy, three with inadequate echocardiographic image quality, one with hypertension, and two with incomplete clinical data), leaving 33 for the final analysis. The cohort included 16 patients with systemic lupus erythematosus [SLE; ACR 1997 criteria (9)], 5 with antiphospholipid syndrome [APS; 2006 international consensus (10)], 2 with rheumatoid arthritis [RA; ACR/EULAR 2010 criteria (11)], 2 with Sjögren' s syndrome [SS; AECG 2002 criteria (12)], 2 with systemic sclerosis [SSc; ACR 1980 criteria (13)], and 6 with mixed connective tissue disease (MCTD). Patients were 26–41 years of age [median 32 (IQR 30–34)] and in second trimester [median 23 weeks (IQR 21–23)], with disease duration of 1–14 years [median 4 years (IQR 3–5)]. All AD pregnant patients were receiving maintenance therapy in accordance with the guidelines. Women with SLE, SS, or MCTD received hydroxychloroquine (HCQ, 100 mg twice daily) plus low-dose prednisone (≤10 mg once daily). Pregnant women with APS received HCQ (100 mg twice daily) and aspirin (50–100 mg once daily); some also received methylprednisolone (4 mg once daily) during the first trimester. RA pregnant patients were treated with subcutaneous certolizumab pegol (200 mg every 2 weeks) and oral methylprednisolone (4 mg once daily). Pregnant women with SSc received low-dose prednisone (≤10 mg once daily) and azathioprine (1.5 mg/kg per day). Routine laboratory markers of systemic inflammation (e.g., CRP, ESR, and complement levels) and formal disease activity indices (e.g., SLEDAI, BVAS, and DAS28) were not systematically available at the time of echocardiographic examination. All patients were asymptomatic at the time of enrollment and were considered clinically stable by their treating rheumatologists at enrolment.

In the AD group, thirty non-pregnant women with AD were recruited during the same period. Four were excluded (one with disease flare, one with chronic kidney disease, and two with valvular heart disease), leaving 26 patients. Age and disease duration were frequency-matched to the AD-P group. Diagnoses comprised 16 SLE, 2 APS, 3 RA, 2 SSc, 1 SS, and 2 MCTD. Patients were 24–41 years of age [median 30 (IQR 25–36)] with disease duration of 1–13 years [median 5 years (IQR 2–7)]. All were on stable medication regimens, generally at slightly higher doses than those used in pregnant patients. The collection of clinical data in AD group was the same as that in AD-P group. All patients were considered clinically stable by their treating rheumatologists at enrolment.

In the H-P group, forty-one healthy women in the second trimester undergoing routine antenatal examination were screened. Four were excluded (two with poor image quality and two with twin pregnancies), leaving 37 participants. Age and gestational age were frequency-matched to the AD-P group. Participants were 26–43 years of age [median 30 (IQR 28–33)] and in second trimester [median 21 weeks (IQR 17–24)]. None had hypertensive disorders or preeclampsia at enrollment.

**Figure 1:**
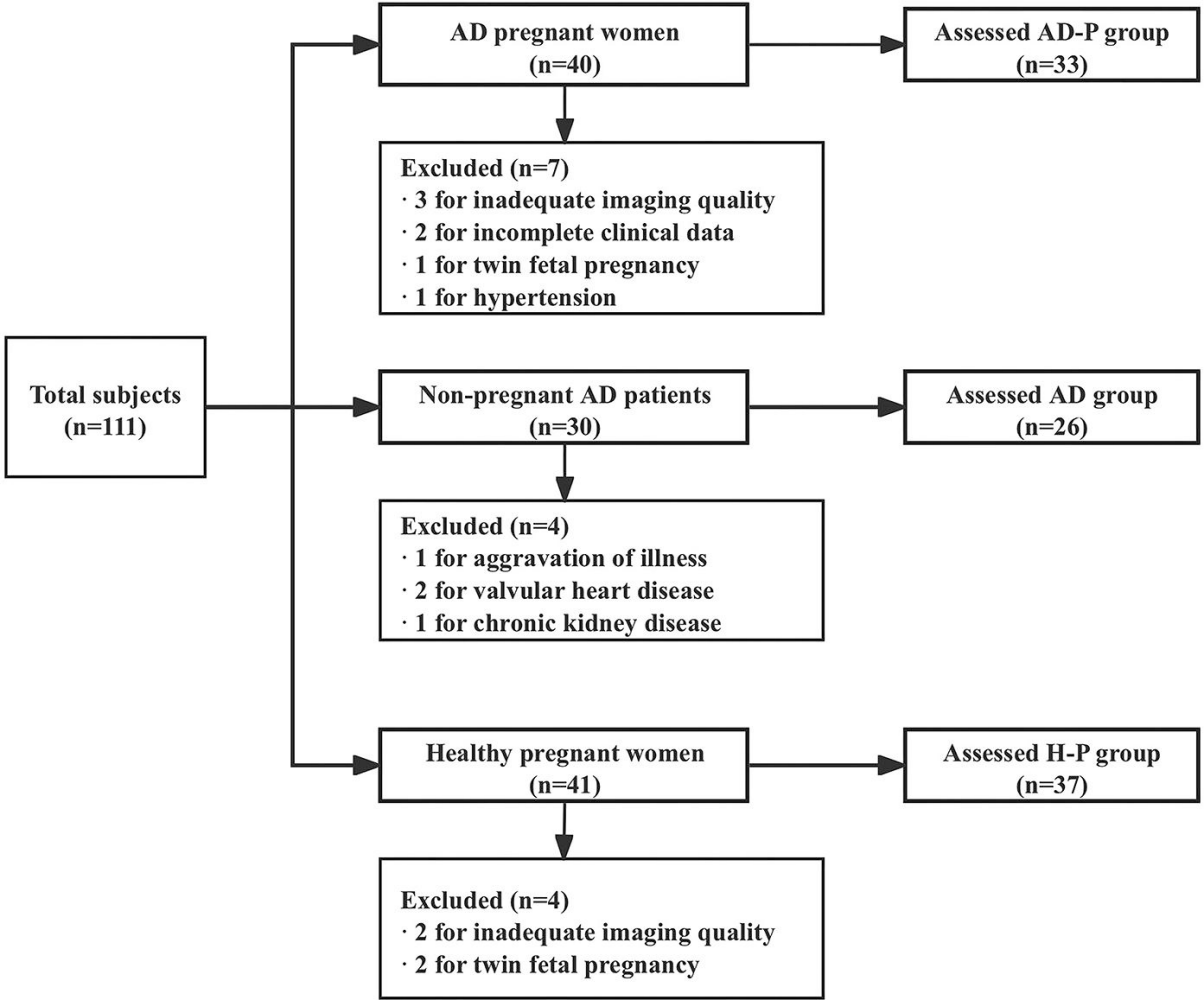
Flowchart of participants enrollment and exclusion.

General exclusion criteria for all groups were: congenital or acquired structural or valvular heart disease (moderate or greater stenosis/regurgitation on echo, history of valve surgery, or congenital valve malformation); ischemic cardiomyopathy; pregnancy complications or twin gestation; hypertension or preeclampsia; abnormal electrocardiogram (atrial fibrillation/flutter, frequent ectopy requiring treatment, bundle branch block/high-grade AV block, or ischemic ST-T changes); history of smoking or alcohol abuse; chronic hepatic or renal disease; incomplete clinical data; unstable disease activity at enrollment; and inadequate echocardiographic image quality. The enrollment flowchart is shown in Figure 1.

### 2.4 Instruments and methods

Instruments: All echocardiographic images were acquired using GE Vivid E9 and E95 ultrasound systems equipped with an S5-1 transducer (frequency range 1.7–3.3 MHz).

Image acquisition and conventional echocardiography: Brachial artery blood pressure was measured three times in the left arm using an electronic sphygmomanometer with the patient at resting supine position, and the average value was recorded. Standard transthoracic echocardiographic was then performed with simultaneous ECG monitoring, following the recommendations of the American Society of Echocardiography (14). Two-dimensional grayscale cine loops were obtained from parasternal long-axis and pical views. The images should be clear and capable of clearly distinguishing the endocardial and epicardial boundaries. Only images with a heart rate variability <5% and frame rates >60 frames/s were accepted for analysis. Conventional parameters included left ventricular end-diastolic and end-systolic diameters (LVDd, LVDs), left ventricular end-diastolic and end-systolic volumes (LVEDV, LVESV), and LVEF calculated by the biplane Simpson method. Left atrial volume index (LAVI) was calculated after correction for body surface area. Mitral inflow E and A waves were obtained by pulsed-wave Doppler to derive the E/A ratio. Tissue Doppler imaging of the septal and lateral mitral annulus was used to measure e’ velocity and the E/e’ ratio was calculated. The left ventricular Tei index was derived from tissue Doppler recordings.

Speckle-tracking and myocardial work analysis: Offline analysis was performed using EchoPAC version 203 (GE Vingmed Ultrasound AS). In apical three-, two-, and four-chamber views, endocardial and epicardial borders were automatically tracked and manually adjusted when necessary to obtain global longitudinal strain (GLS) and peak strain dispersion (PSD). For myocardial work (MW) analysis, the apical three-chamber view was used. The left ventricular mitral valve level, papillary muscle level and apical level were respectively defined as the basal segment, intermediate segment and apical segment. The cardiac cycle was defined according to mitral and aortic valve opening and closure in conjunction with the ECG. Brachial blood pressure values were entered to construct PSLs and derive MW parameters. Global MW parameters included global work index (GWI), global constructive work (GCW), global wasted work (GWW), and global work efficiency (GWE), calculated as GWE = GCW/(GCW + GWW). Segmental MW indices-work index (WI), constructive work (CW), wasted work (WW), and work efficiency (WE)-were obtained for basal, mid, and apical LV segments.

### 2.5 Reproducibility analysis

Ten participants were randomly selected for assessment of inter- and intra-observer reproducibility. Left ventricular strain and myocardial work parameters were analyzed independently by two senior echocardiographers. One observer repeated the analysis after a 7 day interval. Agreement was evaluated using Bland-Altman and the intraclass correlation coefficient (ICC), which was interpreted as excellent for ICC ≥0.80, good for 0.60 ≤ ICC < 0.80, fair for 0.40 ≤ ICC < 0.60, and poor for ICC < 0.40.

### 2.6 Statistical analysis

All data were analyzed using SPSS version 25.0 (IBM Corp., Armonk, NY, USA). The Shapiro–Wilk test was applied to assess the normality of continuous variables. Normally distributed data are expressed as mean ± standard deviation, and non-normally distributed data as median (interquartile range). Categorical variables are expressed as counts and percentages. Between-group comparisons were conducted using the independent-samples *t*-test or Mann–Whitney *U*-test for continuous variables and the chi-square test or Fisher's exact test for categorical variables, as appropriate. Analyses of covariance (ANCOVA) were used to determine differences in MW parameters after incorporation of covariate parameters. Model A adjusted for age, SBP, heart rate, and disease duration. Model B adjusted for age, SBP, heart rate, and multiparity. Covariates were pre-specified based on clinical relevance and potential group imbalance. Before fitting ANCOVA and interaction models, we evaluated multicollinearity among covariates using variance inflation factors (VIFs). All VIF values were <2.0, indicating low multicollinearity. Group × SBP and Group × disease duration interaction terms were specified *a priori* based on biological plausibility, and significant interactions were explored using simple-slope analyses. For each outcome, adjusted mean differences (Δ) between groups and corresponding 95% confidence intervals (CI) were reported. Multiple testing was controlled using the Benjamini–Hochberg false discovery rate (FDR) procedure, with a two-sided α of 0.05. Correlations were assessed using Pearson or Spearman correlation analyses, as appropriate. Partial correlation analysis was used to evaluate associations between MW parameters and clinical variables while controlling for potential confounders. A two-tailed *P* value < 0.05 was considered statistically significant.

## 3 Results

### 3.1 Comparison of baseline clinical characteristics

In the AD-P vs. AD group comparison, there were no significant differences in age, body surface area (BSA), heart rate, blood pressure, or disease duration. However, the proportion of patients positive for anti-SSA/Ro and anti-SSB/La antibodies was significantly higher in the AD-P group than in the AD group (both *P* < 0.05).

In the AD-P vs. H-P group comparison, age, BSA, heart rate, blood pressure, and gestational age were similar between groups. All pregnant participants were in the second trimester. The proportion of women with a history of multiple pregnancies was significantly higher in the AD-P group than in the H-P group (*P* < 0.05). Baseline characteristics are summarized in Table 1.

**Table 1:** Baseline clinical characteristics.

Clinical data	AD-P group (*n* = 33)	AD group (*n* = 26)	H-P group (*n* = 37)
General clinical parameters			
Age (years)	32 (30–34)	30 (25–36)	30 (28–33)
BSA (m^2^)	1.63 (1.56–1.75)	1.57 (1.52–1.69)	1.65 (1.51–1.72)
Heart rate (bpm)	84 ± 7	87 ± 15	86 ± 11
Systolic BP (mmHg)	113 (109–122)	114 (109–126)	113 (107–121)
Diastolic BP (mmHg)	71 (66–78)	75 (70–78)	70 (68–76)
Gestational age (weeks)	23 (21–23)	-	21 (17–24)
Multiparity, *n* (%)	20 (61%)*	10 (38%)	2 (5%)
Disease duration (years)	5 (2–7)	4 (3–5)	–
Antibody detection			
Anticardiolipin positive *n* (%)	7 (21%)	6 (23%)	–
Anti-SSA/Ro positive, *n* (%)	19 (58%)+	6 (23%)	–
Anti-SSB/La positive, *n* (%)	9 (27%)+	1 (3%)	–
Anti-nRNP positive, *n* (%)	6 (18%)	4 (15%)	–
Medication situation			
Hydroxychloroquine (HCQ), *n* (%)	29 (87%)	21 (81%)	–
Prednisone, *n* (%)	26 (79%)	19 (73%)	–
Aspirin, *n* (%)	5 (15%)	2 (8%)	–
Methylprednisolone, *n* (%)	5 (15%)	5 (19%)	–
Certolizumab, *n* (%)	2 (6%)	3 (12%)	–
Azathioprine, *n* (%)	2 (6%)	2 (8%)	–

* *P* < 0.05 vs. H-P.

+ *P* < 0.05 vs. AD.

### 3.2 Comparison of conventional echocardiographic parameters

In the AD-P vs. AD group comparison, LVEDV and LVESV were significantly higher in the AD-P group (both *P* < 0.05), whereas LVEF and the Tei index did not differ significantly.

In the AD-P vs. H-P group comparison, LVEDV, LVESV, and E/e’ ratio were significantly higher and E/A ratio was significantly lower in the AD-P group (all *P* < 0.05), while LVEF and the Tei index remained comparable. Conventional echocardiographic findings are shown in Table 2.

**Table 2:** Conventional echocardiographic parameters.

Parameters	AD-P group (*n* = 33)	AD group (*n* = 26)	H-P group (*n* = 37)
LVDd (mm)	45.00 (42.50, 47.00)	45.00 (42.70, 48.00)	44.00 (42.00, 47.00)
LVDs (mm)	29.12 ± 3.17	28.62 ± 2.87	28.19 ± 2.48
LVMI (g/m^2^)	132.14 (113.42, 152.36)	133.34 (119.578, 152.91)	143.98 (105.60, 162.51)
LVEDV (mL)	79.48 ± 15.56*+	69.65 ± 15.80	70.86 ± 17.68
LVESV (mL)	30.00 (24.50, 35.00)*+	26.50 (17.00, 37.25)	24.00 (19.00, 31.50)
Systolic function			
LVEF (%)	62.55 ± 5.17	63.63 ± 7.55	64.89 ± 5.38
Diastolic function			
E/A	1.44 (1.20, 1.66)*	1.31 (0.74, 1.61)	1.71 (1.42, 2.09)
E/e′	6.64 (5.54, 7.70)*	6.54 (5.97, 7.35)	5.66 (5.14, 6.47)
LAVI (mL/m^2^)	26.29 (20.56, 32.22)	25.39 (20.23, 31.16)	25.63 (18.80, 30.59)
Global function			
Tei index	0.39 (0.33, 0.44)	0.41 (0.36, 0.45)	0.36 (0.33, 0.40)

* *P* < 0.05 vs. H-P.

+ *P* < 0.05 vs. AD.

### 3.3 Comparison of two-dimensional strain and myocardial work parameters

In multivariable models, there was no evidence of problematic multicollinearity among covariates (all VIF <2.0).

**Figure 2:**
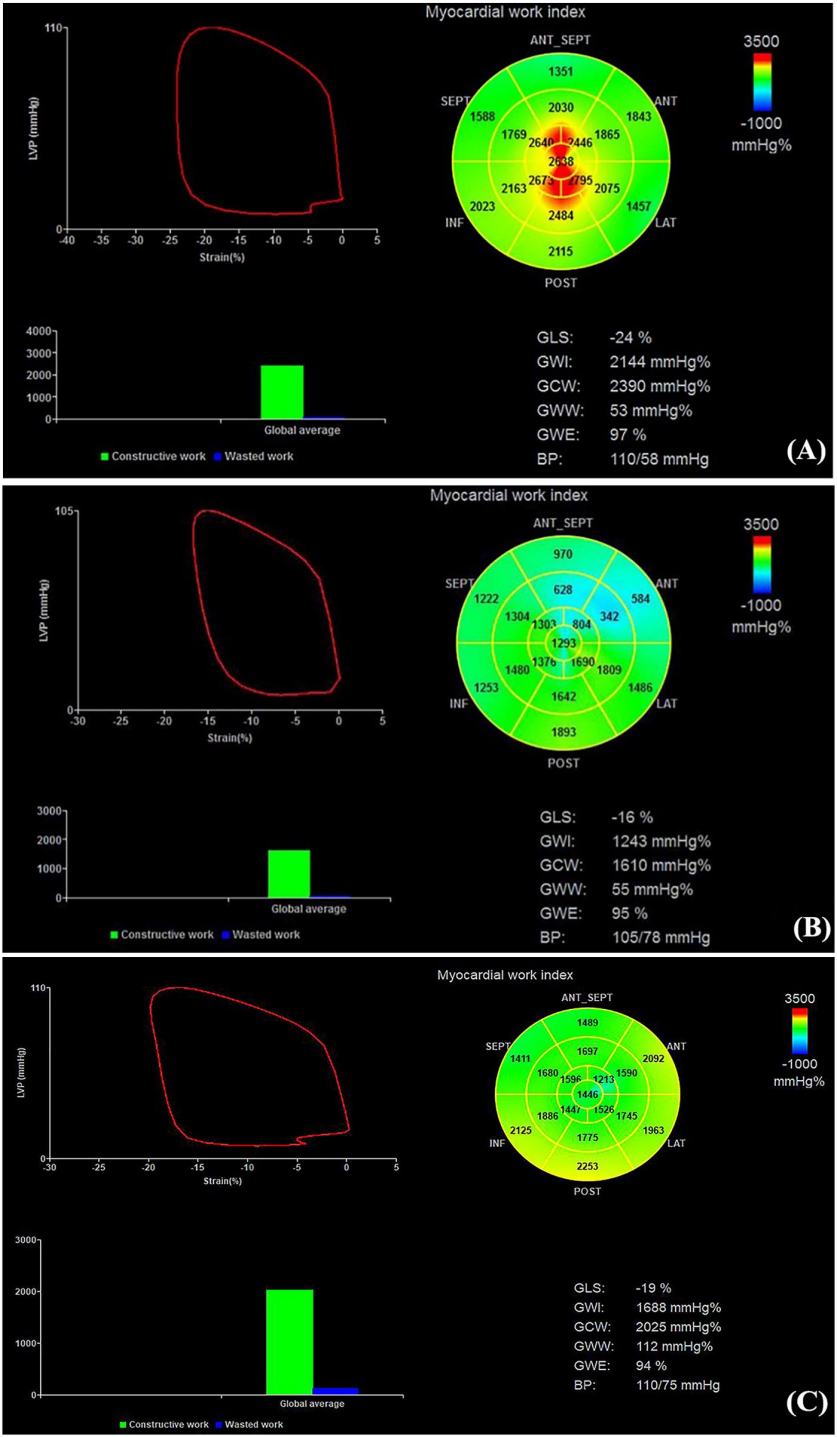
Overview of myocardial work parameters. Top left: left-ventricular pressure-strain loop (PSL); the loop area represents the global work index (GWI). Top right: 17-segment LV myocardial work bull's-eye plot. Bottom left: bar chart of global constructive work (GCW) and global wasted work (GWW). Bottom right: global longitudinal strain (GLS), global myocardial work metrics, and blood pressure. **(A–C)** are the representative myocardial work analysis diagrams of the H-P, AD-P, and AD groups respectively.

**Figure 3:**
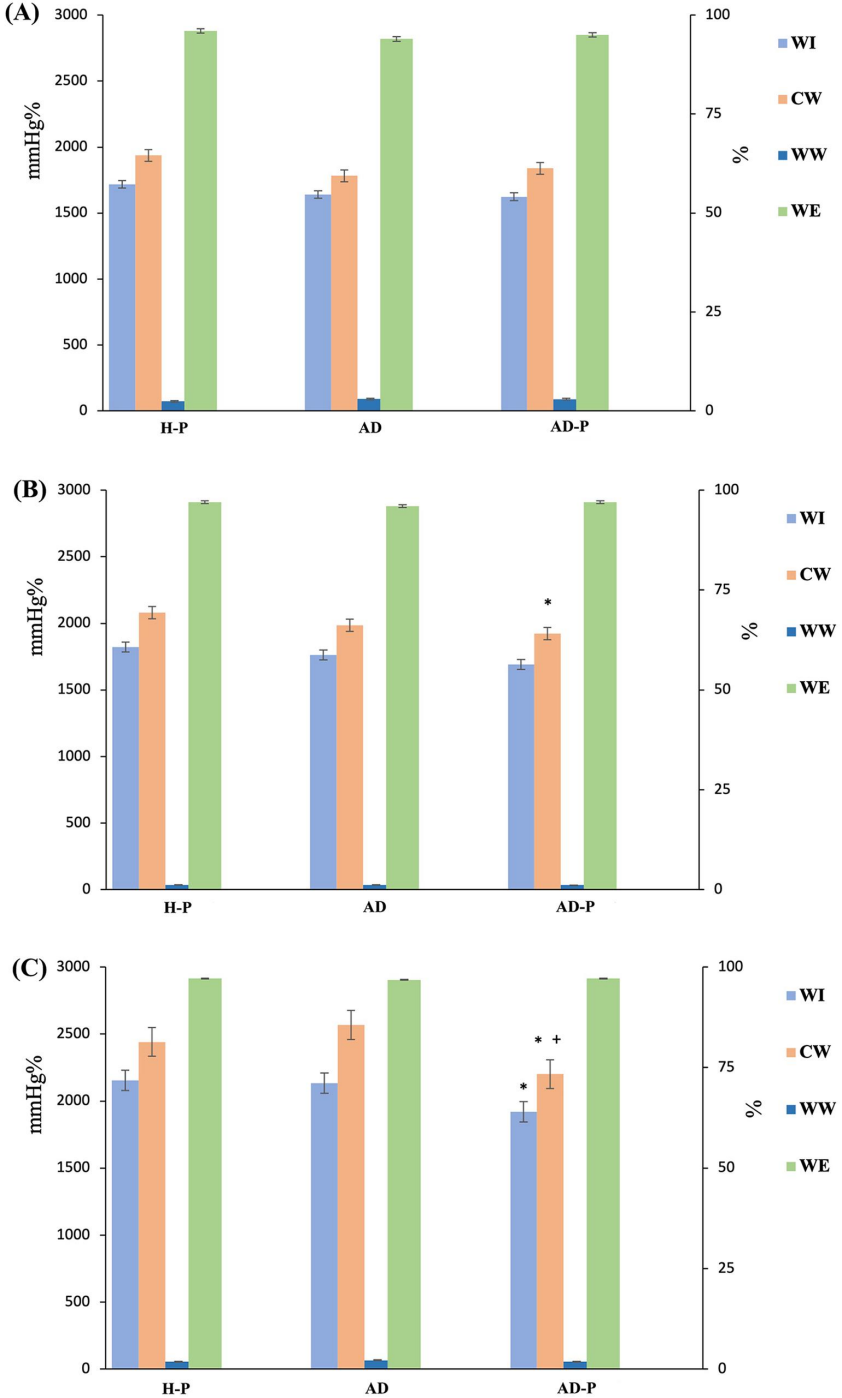
Segmental myocardial work across groups before ANVOCA. Bar charts compare segmental myocardial work between the AD-P group and the other two groups (H-P and AD). Versus H-P, the AD-P group shows significantly lower apical WI and CW, as well as lower mid-segment CW. Versus AD, apical CW is also significantly reduced in AD-P. **(A)**, basal segment; **(B)**, mid segment; **(C)**, apical segment. Statistics: *P < 0.05 vs. H-P, ^+^*P* < 0.05 vs. AD.

**Figure 4:**
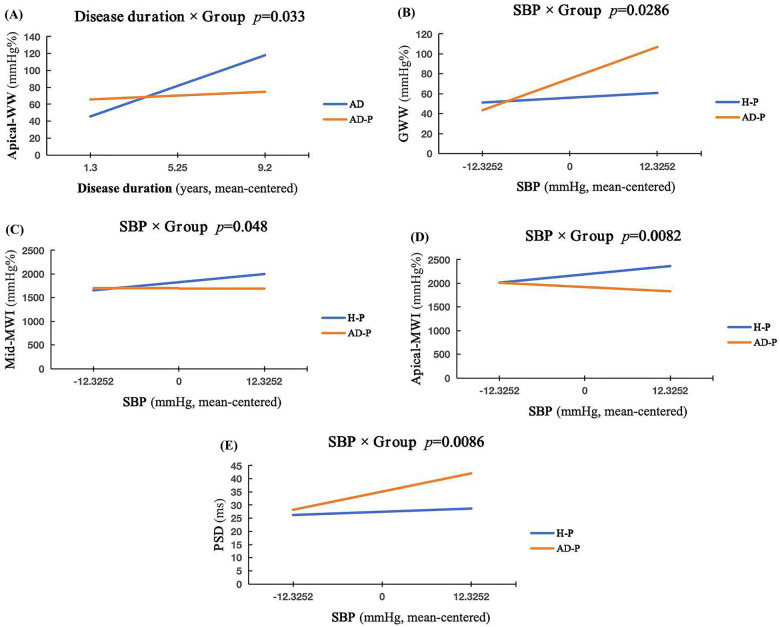
Simple slope plots of the interaction parameters for AD-P vs. AD and AD-P vs. H-P. **(A)**, AD-P vs. AD, Group × Disease duration interaction in apical-WW. AD slope = 9.31/year [*P* < .001; 95% CI (4.76, 13.86)], AD-P slope = 1.16/year [*P* = 0.74; 95% CI (−15.61, −0.69)]. Interaction: B = −8.15/year, *P* = 0.033. **(B)**, AD-P vs. H-P, Group × SBP interaction in GWW. H-P slope = 0.392/mmHg [*P* = 0.580; 95% CI (−1.012, 1.796)]; AD-P slope = 2.572/mmHg [*P* = 0.0003; 95% CI (1.225, 3.920)]. Interaction: B = 2.181/mmHg, *P* = 0.0286. **(C)**, AD-P vs. H-P, Group × SBP interaction in mid-WI. H-P slope = 14.15/mmHg [*P* = 0.015; 95% CI (2.82, 25.48)]; AD-P slope = −0.70/mmHg [*P* = 0.840; 95% CI (−18.15-3.59)]. Interaction: B = −14.60/mmHg, *P* = 0.0048. **(D)**, AD-P vs. H-P, Group × SBP interaction in apical-WI. H-P slope = 14.15/mmHg [*P* = 0.015; 95% CI (2.82, 25.48)]; AD-P slope = −7.28/mmHg [*P* = 0.186; 95% CI (−18.15, 3.59)]. Interaction: B = −21.43/mmHg, *P* = 0.0082. **(E)**, AD-P vs. H-P, Group × SBP interaction in PSD. H-P slope = 0.099/mmHg [*P* = 0.423; 95% CI (−0.147, 0.346)]; AD-P slope = 0.562/mmHg [*P* < 0.001; 95% CI (0.326, 0.798)]. Interaction: B = 0.463/mmHg, *P* = 0.0086.

In the AD-P vs. AD group comparison, GLS, PSD, and global MW indices did not differ significantly between groups (all *P* > 0.05). However, apical-CW was significantly reduced in the AD-P group (*P* < 0.05) (Table 3 and Figures 2, 3). In ANCOVA Model A, apical-CW remained lower in the AD-P group after adjustment for age, SBP, heart rate, and disease duration (FDR-adjusted *P* = 0.036). Apical-WW showed a significant Group × disease-duration interaction, indicating that the association between disease duration and apical-WW differed between pregnant and non-pregnant patients with AD. Exploratory simple-slope analyses suggested that this relationship was more pronounced in the AD group. Details are presented in Table 4 and Figure 4.

**Table 3:** GLS, PSD and myocardial work parameters.

Parameters	AD-P group (*n* = 33)	AD group (*n* = 26)	H-P group (*n* = 37)
GLS (%)	−19.00 (−22.00, −16.00)*	−20.00 (−22.25, −18.00)	−21.00 (−22.00, −20.00)
PSD (ms)	32 (24, 44)*	32 (25,44)	27 (23, 32)
GWI (mmHg%)	1,756 ± 310*	1,864 ± 355	1,901 ± 254
GCW (mmHg%)	2,004 ± 313*	2,125 ± 347	2,157 ± 252
GWW (mmHg%)	58 (43, 82)	63 (49, 103)	55 (35, 74)
GWE (%)	96 (94, 97)*	96 (95, 97)	97 (96, 98)

* *P* < 0.05 vs. H-P.

**Table 4:** Adjusted ANCOVA results of model A.

MW parameters	Δ	95% CI for Δ	*P*	FDR- *P*
Global myocardial work				
** **GWE	0.111	−1.594, 1.372	0.881	0.982
** **GWI	−129.424	−303.808, −44.96	0.142	0.554
** **GCW	−150.872	−319.957, 18.214	0.079	0.474
** **GWW	−0.542	−32.732, 31.649	0.973	0.982
Regional myocardial work				
** **basal-WI	−34.720	−199.411, 129.970	0.674	0.982
** **basal**-**WE	0.742	−1.520, 3.004	0.513	0.982
** **basal**-**CW	28.586	−136.701, 193.873	0.730	0.982
** **basal**-**WW	−0.991	−47.703, 45.722	0.966	0.982
** **mid**-**WI	−168.311	−320.358, 16.264	0.311	0.800
** **mid**-**WE	−0.176	−1.773, 1.421	0.825	0.982
** **mid**-**CW	−97.085	−275.429, 81.260	0.279	0.360
** **mid**-**WW	7.892	−27.012, 42.796	0.652	0.982
** **apical**-**WI	−252.234	−504.033, −0.434	0.04	0.360
** **apical**-**WE	0.026	−1.258, 1.309	0.968	0.982
** **apical**-**CW	−414.285	−669.145, −159.424	0.002	0.036
** **apical**-**WW^a^	−11.824	−40.026, 16.378	0.404	0.909
Strain indices				
** **GLS	1.173	−0.454, 2.800	0.154	0.554
** **PSD	0.069	−6.173, 6.311	0.982	0.982

Model A adjusted for age, systolic blood pressure (SBP), heart rate, and disease duration. Levene's test was performed for homogeneity of variance. When heteroscedasticity was present, HC3 heteroscedasticity-robust standard errors and *P* values were reported. Otherwise, conventional ANCOVA inference (standard) was used. Δ, adjusted mean difference.

a Significant interaction with disease duration (Group × disease duration).

In the AD-P vs. H-P group comparison, the AD-P group had significantly higher PSD and lower GLS, GWI, GCW, and GWE (all *P* < 0.05). GWW did not differ significantly between groups (*P* > 0.05). In addition, apical-WI, apical-CW, and mid-CW were significantly lower in the AD-P group (all *P* < 0.05) (Table 3 and Figures 2, 3). In ANCOVA Model B, GWI, GCW, GWE, and apical-CW remained lower in the AD-P group (FDR-adjusted *P* = 0.022, 0.009, 0.022, 0.043, respectively), and PSD remained higher (FDR-adjusted *P* < 0.001) after adjustment for age, SBP, heart rate, and multiparity. GWW, mid-WI, apical-WI, and PSD showed significant Group × SBP interaction, indicating that the relationship between SBP and these MW indices differed between AD-P and H-P. Exploratory simple-slope analyses suggested that this relationship was more pronounced in the AD-P group. These results are summarized in Table 5 and Figure 4.

**Table 5:** Adjusted ANCOVA results of model B.

MW parameters	Adjusted mean difference (Δ)	95% CI for Δ	*P*	FDR- *P*
Global myocardial work				
GWE	−1.457	−2.543, −0.485	0.005	0.022
GWI	−223.138	−374.421, −71.856	0.005	0.022
GWI	−249.696	−392.837, −106.554	0.001	0.009
GWW^‡^	27.438	−1.424, 56.301	0.062	0.101
GCW	−126.674	−269.662, 16.31.	0.081	0.112
GWW	−1.017	−2.673, 0.639	0.224	0.259
Regional myocardial work				
basal-WI	−155.269	−294.646, −15.892	0.030	0.070
basal-WE	25.056	−16.996, 67.108	0.238	0.259
basal-CW^‡^	−168.311	−320.358, −16.264	0.031	0.070
basal-WW	−0.936	−2.125, 0.252	0.245	0.259
mid-WI	−125.023	−347.958, 67.433	0.04	0.080
mid-WE	14.783	−16.780, 46.347	0.383	0.383
mid-CW^‡^	−331.137	−571.243, −91.032	0.08	0.112
mid-WW	−1.181	−2.411, 0.049	0.060	0.101
apical-WI	−224.145	−573.986, −73.335	0.012	0.043
apical-WE	19.690	−18.387, 56.781	0.151	0.194
apical-CW	2.207	−0.081, 3.797	0.02	0.060
apical-WW^‡^	10.136	5.058, 15.214	0.000	< 0.001
Strain indices				
GLS	2.207	−0.081 to 3.797	0.02	0.060
PSD	10.136	5.058, 15.214	0.000	< 0.001

Model B adjusted for age, systolic blood pressure (SBP), heart rate, and multiparity. Levene's test was performed for homogeneity of variance. When heteroscedasticity was present, HC3 heteroscedasticity-robust standard errors and *P* values were reported. Otherwise, conventional ANCOVA inference (Standard) was used. Δ, adjusted mean difference.

^‡^Significant interaction with SBP (Group × SBP).

### 3.4 Correlation analysis

In the AD-P group, univariate correlation analysis showed that disease duration was positively correlated with GLS (*r_s_* = 0.361, *P* = 0.020), and anti-SSA/Ro antibody positivity was positively correlated with PSD *(r* = 0.416, *P* = 0.016). Parity was positively correlated with GCW (*r_s_* = 0.408, *P* = 0.018). Disease duration was negatively correlated with GWE (*r_s_* = −0.510, *P* = 0.002) and positively correlated with GWW (*r_s_* = 0.485, *P* = 0.004) (Table 6). In partial correlation analysis, after controlling the variables, PSD was positively correlated with anti-SSA/Ro antibody positivity (*r* = 0.379, *P* = 0.039), and GWW was positively correlated with anti-SSB/La antibody positivity (*r* = 0.419, *P* = 0.029). However, no significant correlations were found between MW parameters and either disease duration or multiparity (Table 7).

**Table 6:** Correlations of GLS, PSD, and MW parameters with clinical variables in the AD-P group.

Clinical variables	GLS (%)		PSD (ms)		GWI (mmHg%)		GCW (mmHg%)		GWW (mmHg%)		GWE (%)	
	*r_s_*	*P*	*r*	*P*	*r_s_*	*P*	*r_s_*	*P*	*r_s_*	*P*	*r_s_*	*P*
Parity (number of pregnancies)	−0.199	0.211	−0.177	0.325	0.287	0.105	0.408	0.018	−0.077	0.672	−0.019	0.916
Disease duration (years)	0.361	0.020	0.319	0.071	−0.320	0.069	−0.115	0.524	0.485	0.004	−0.510	0.002
Anticardiolipin positive	−0.120	0.453	−0.136	0.449	−0.008	0.966	0.039	0.830	0.027	0.880	−0.131	0.469
Anti-SSA/Ro positive	−0.068	0.672	0.416	0.016	−0.208	0.244	−0.332	0.059	0.156	0.385	−0.225	0.208
Anti-SSB/La positive	0.111	0.488	−0.071	0.694	−0.147	0.416	−0.118	0.513	−0.104	0.566	0.080	0.658
Anti-nRNP positive	−0.187	0.241	0.007	0.969	0.281	0.114	0.248	0.165	−0.260	0.144	0.285	0.107

*r_s_* = Spearman's rho; *r* = Pearson's correlation coefficient.

**Table 7:** Partial correlation analysis between GLS, PSD, and MW parameters and clinical data in AD-P group.

Control variables	Variable	GLS (%)	PSD (ms)	GWI (mmHg%)	GCW (mmHg%)	GWE (%)	GWW (mmHg%)
Gestational age & disease duration & multiparity & age & SBP& heart rate	Anti-SSA/Ro positive	*r* = 0.379	*r* = 0.378	*r* = −0.345	*r* = −0.352	*r* = −0.236	*r* = 0.165
		*P* = 0.051	*P* = 0.039	*P* = 0.062	*P* = 0.057	*P* = 0.208	*P* = 0.384
	Anticardiolipin positive	*r* = 0.086	*r* = −0.098	*r* = 0.034	*r* = −0.028	*r* = −0.019	*r* = −0.081
		*P* = 0.650	*P* = 0.608	*P* = 0.859	*P* = 0.885	*P* = 0.923	*P* = 0.676
	Anti-SSB/La positive	*r* = 0.100	*r* = 0.119	*r* = −0.113	*r* = −0.020	*r* = −0.365	*r* = 0.419
		*P* = 0.597	*P* = 0.523	*P* = 0.551	*P* = 0.917	*P* = 0.062	*P* = 0.029
Gestational age & multiparity & age & SBP & heart rate	Disease duration	*r* = 0.206	*r* = −0.010	*r* = −0.231	*r* = −0.098	*r* = −0.156	*r* = 0.105
		*P* = 0.293	*P* = 0.962	*P* = 0.238	*P* = 0.620	*P* = 0.429	*P* = 0.597
Gestational age & disease duration & age & SBP & heart rate	Multiparity	*r* = −0.244	*r* = −0.221	*r* = 0.213	*r* = 0.301	*r* = 0.077	*r* = −0.119
		*P* = 0.258	*P* = 0.211	*P* = 0.277	*P* = 0.120	*P* = 0.699	*P* = 0.547

### 3.5 Reproducibility

**Figure 5:**
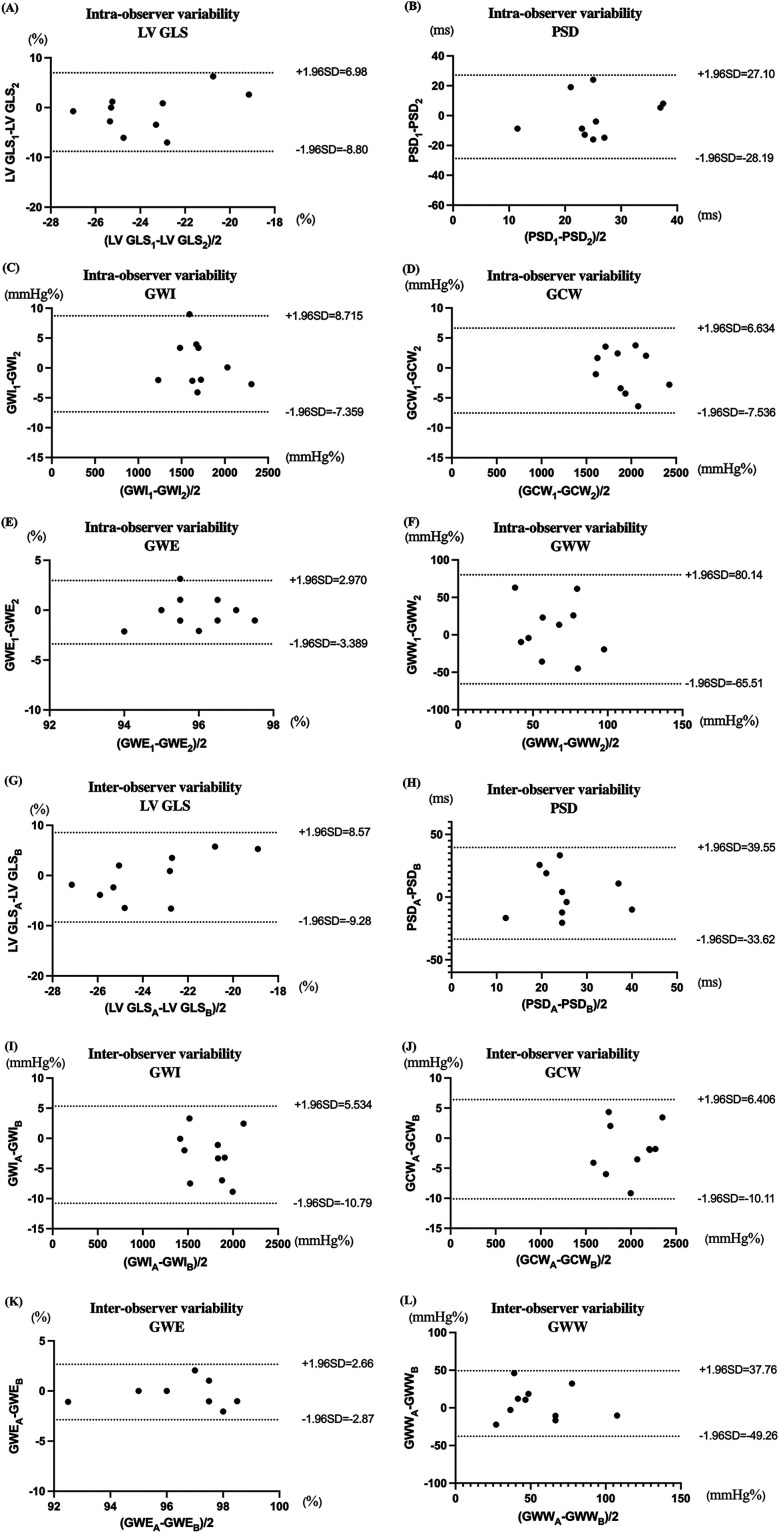
Bland-Altman plot analysis for intra-observer and inter-observer variability for LV GLS, PSD, GWI, GCW, GWE, and GWW. (Parameter)_1_ and (Parameter)_2_ respectively represent the first and second measurements conducted by the same echocardiographer. (Parameter)_A_ represents measure performed by the echocardiographer A. (Parameter)_B_ represents measure performed by the echocardiographer B. **(A–F)** represent the Bland-Altman plot analysis for intra-observer for LV GLS, PSD, GWI, GCW, GWE, and GWW, respectively. Panels: **(G–L)** represent the Bland-Altman plot analysis for inter-observer for LV GLS, PSD, GWI, GCW, GWE, and GWW, respectively.

Intraclass correlation coefficient analysis demonstrated good intra- and inter-observer reproducibility, with *ICC* values >0.80 for LV GLS, PSD, GWI, GCW, GWW, and GWE. Detaile *ICC v*alues and Bland-Altman plots are provided in Table 8 and Figures 5.

**Table 8:** ICC values of intra-observer and inter-observer.

Intra-observer	*ICC*	*P*	Inter-observer	*ICC*	*P*
LV GLS	0.929	<0.001	LV GLS	0.919	<0.001
PSD	0.895	<0.001	PSD	0.863	<0.001
GWI	0.963	<0.001	GWI	0.947	<0.001
GCW	0.961	<0.001	GCW	0.928	<0.001
GWW	0.879	<0.001	GWW	0.847	<0.001
GWE	0.973	<0.001	GWE	0.802	0.01

## 4 Discussion

In this study, we attempted to characterize the left ventricular mechanical features of pregnant women with autoimmune diseases (AD) by combining conventional echocardiography with myocardial work (MW) analysis. Comparing these patients with non-pregnant AD cohorts and healthy pregnant controls, the main findings can be summarized as follows: (i) Despite the preservation of LVEF, AD pregnant women (AD-P) exhibited a larger left ventricular volume and diastolic dysfunction. (ii) The global MW parameters of AD-P were slightly lower than those of the healthy pregnancy control group, while approximately similar to those of non-pregnant AD patients. (iii) Compared with the other two groups, the apical-CW of AD-P patients selectively decreased, indicating a regional exacerbation pattern of subclinical dysfunction.

Considering the hemodynamic burden during pregnancy, the changes in LV volume in such patients are reasonable. When evaluating cardiac function, we found that its diastolic function was relatively reduced while the LVEF was retained. Therefore, we used LV PSL technique to obtain MW parameters to further assess its systolic function and identify whether there was subclinical damage.

In our analysis of MW parameters, global MW did not differ significantly between AD-P and AD patients. Compared with healthy pregnant women, AD-P patients had significantly lower GLS, GWI, GCW, and GWE. Considering clinical variables may influence between-group differences, we used ANCOVA to adjust for key confounders. After adjustment, global MW parameters remained similar between AD-P and AD patients, whereas AD-P patients still showed lower GWI, GCW, and GWE compared with H-P. In healthy adults, reference values for GWI and GCW are approximately 1,900–2,100 mmHg% and 2,200–2,500 mmHg%, respectively, and with GWE around 92%–96% (15). Relative to these reference ranges, the median values in AD-P indicate a tendency toward lower LV myocardial work. Our results are also consistent with previous PSL studies in autoimmune and pregnancy-related conditions, in which GWE reduced despite preserved LVEF (16, 17). Moreover, Olsen et al. suggests that even modest changes in GWI and GCW may signal subclinical myocardial injury and aid risk stratification (18, 19). Longitudinal studies are therefore needed to determine whether these relatively small differences in MW parameters translate into adverse clinical events.

The most important and novel observation was the consistent reduction of apical-CW in AD-P, whereas basal and mid-segmental MW were relatively preserved. This apical performence is physiologically plausible. The LV apex is supplied by distal coronary branches and is considered particularly vulnerable to disturbances in microvascular perfusion and wall stress, both of which are frequently altered in autoimmune diseases (20). Chronic immune-mediated myocardial involvement may therefore preferentially affect the distal ventricular myocardium, creating a functional “weakest link” at the apex (16). Superimposed pregnancy-related increases in blood volume and heart rate further augment wall stress and oxygen demand in this region (21, 22). Thus, when the hemodynamic burden of pregnancy is added to an already vulnerable myocardium, the apex may be the first segment to exhibit reduced constructive work while global MW parameters remain within the normal range. Taken together, our data suggest that apical-CW may capture an early stage of regionally accentuated dysfunction that is not yet reflected in global parameters and serve as a practical regional target for monitoring early myocardial dysfunction.

Our exploratory interaction analyses suggested that pregnancy-related cardiovascular adaptation and treatment adjustment may weaken the association between cumulative disease duration and apical-WW. The same analyses also identified SBP as an important modifier of MW in AD-P, with higher SBP associated with greater mechanical dyssynchrony. These observations support the potential value of careful afterload control in this population.

After adjustment, AD-P patients still showed higher PSD compared with H-P. The concomitant increase in PSD reflects greater LV mechanical dispersion (23), indicating subclinical mechanical dyssynchrony in AD-P that is more likely related to underlying disease-associated myocardial involvement than to pregnancy-related loading conditions alone. Prior work in autoimmune cohorts supports the notion that immune-mediated cardiac remodelling underlies such dispersion abnormalities (24), and have linked anti-SSA antibodies to conduction system damage. This is not only seen in fetuses at risk of congenital atrioventricular block, but also in adults with atrioventricular block and arrhythmias (25–27). In our cohort, PSD showed a weak positive association with anti-SSA/Ro positivity after multivariable adjustment. Given the modest correlation strength, larger multicenter cohorts will be required to confirm these relationships and clarify their prognostic value.

This study has several limitations. First, this is a single-center, small-sample, and cross-sectional study lacking long-term follow-up. Second, we did not separately analyze the impact of specific drugs, inflammatory indicators, and disease activity indices on cardiac function. Third, our cohort includes multiple autoimmune entities, which may introduce disease heterogeneity. Larger disease-focused cohorts are needed to address subtype issues in future work. Fourth, Fourth, RV structure and function were not comprehensively assessed.

Despite these limitations, our study has several strengths and yields clinically relevant insights. By comparing global and segmented MW parameters among AD pregnant women, non-pregnant AD patients, and healthy pregnant women in a single design, we were able to partially separate disease-related from pregnancy-related effects and to identify an apical-predominant pattern of subclinical dysfunction. Clinically, this may contribute to a more nuanced cardiovascular risk stratification than is achievable with conventional echocardiography and GLS alone. This suggests that pregnant women with autoimmune disease and abnormal MW profiles might merit closer echocardiographic surveillance, more stringent optimization of blood pressure and coordinated care within a multidisciplinary cardio–obstetric team.

## 5 Conclusion

Pregnant women with autoimmune diseases showed reduced mycardial work, while increased levels of left ventricular dyssynchrony in comparison to healthy pregnant women. Quantitative assessment of myocardial work can be used to detect subclinical left ventricular dysfunction in pregnant women with autoimmune diseases. The apical-CW become a new monitoring target cardiac function impairment. which may help clinicians better understand and manage those pregnant women.

## Data Availability

The original contributions presented in the study are included in the article/Supplementary Material, further inquiries can be directed to the corresponding authors.
